# A Meta-Analysis of First-Line Treatments for Unresectable Pleural Mesothelioma: Indirect Comparisons from Reconstructed Individual Patient Data of Six Randomized Controlled Trials

**DOI:** 10.3390/cancers17030503

**Published:** 2025-02-03

**Authors:** Andrea Messori, Sabrina Trippoli, Eugenia Piragine, Sara Veneziano, Vincenzo Calderone

**Affiliations:** 1 HTA Unit, Regione Toscana, 50136 Firenze, Italy; sabrina.trippoli@regione.toscana.it; 2 Department of Pharmacy, University of Pisa, 56126 Pisa, Italy; eugenia.piragine@unipi.it (E.P.); sara.veneziano@phd.unipi.it (S.V.); vincenzo.calderone@unipi.it (V.C.); 3 School of Specialization in Hospital Pharmacy, University of Pisa, 56126 Pisa, Italy

**Keywords:** pleural mesothelioma, pemetrexed, cisplatin, nivolumab+ipilimumab, bevacizumab+pemetrexed+cisplatin, pembrolizumab, durvalumab, reconstruction of patient-level data

## Abstract

Background: In unresectable pleural mesothelioma, pemetrexed+cisplatin as first line is considered the standard of care, but novel treatments have been recently proposed. Methods: Our objective was to compare, albeit indirectly, the results of randomized controlled trials on overall survival (OS). The IPDfromKM method was employed for reconstruct individual patient data (IPD) from the graphs of Kaplan–Meier curves. Cox statistics was run to estimate hazard ratios (HRs). Results: After a literature search on Medline (via PubMed) and Scopus databases, six randomized controlled trials were identified in which five new treatments (nivolumab plus ipilimumab, bevacizumab plus pemetrexed plus cisplatin, chemotherapy plus pembrolizumab, ONCOS-102 plus pemetrexed plus cisplatin/carboplatin and cediranib plus pemetrexed+cisplatin with maintenance with cediranib) were evaluated. In five trials, pemetrexed plus cisplatin was the standard of care given to the control arms. Nivolumab plus ipilimumab, bevacizumab plus pemetrexed plus cisplatin and chemotherapy plus pembrolizumab showed a significantly better OS compared with controls. ONCOS-102 plus pemetrexed plus cisplatin/carboplatin did not significantly improve OS. In contrast, OS worsened with cisplatin alone and with cediranib plus pemetrexed+cisplatin with maintenance with cediranib. Discussion: Our analysis indicates that, in patients with unresectable pleural mesothelioma, three of the five novel treatments provided a significant survival benefit compared with the standard of care. Further research is needed to confirm the OS benefit found in our analysis with some treatments, whereas cisplatin alone and cediranib plus pemetrexed+cisplatin with maintenance with cediranib do not seem to deserve further research.

## Simple Summary

This study focuses on unresectable pleural mesothelioma, a serious disease for which the combination of pemetrexed plus cisplatin is the current standard of care in the first-line setting. In recent years, the proposal of novel first-line treatments has raised the need for a comprehensive comparison of these new therapeutic alternatives. The present study was specifically designed to address this issue. Six randomized controlled trials were identified from PubMed and Scopus and analyzed, considering the overall survival as a unique endpoint. In these trials, five new combination regimens were evaluated (nivolumab plus ipilimumab, bevacizumab plus pemetrexed plus cisplatin, chemotherapy plus pembrolizumab, ONCOS-102 plus pemetrexed plus cisplatin/carboplatin and cediranib plus pemetrexed+cisplatin with maintenance with cediranib). The results of our meta-analysis showed poor efficacy for cisplatin alone and for cediranib-based combinations. By contrast, the other alternatives generally showed a significant improvement in overall survival, but the magnitude of this improvement was limited (around 3 months per patient). In conclusion, although some therapeutic advances have been made in the first-line treatment of this disease, the new treatments demonstrated only a clinically modest prolongation of survival. While these new alternatives could represent a new standard of care, further research is needed into other combination treatments providing a more relevant survival improvement.

## 1. Introduction

Malignant pleural mesothelioma is a rare and aggressive carcinoma associated with a poor prognosis [1,2,3]. The diagnosis of pleural mesothelioma is often delayed because of insidious onset of the disease, which contributes to the inauspicious outcome. In fact, the estimated 5-year survival probability in untreated patients is less than 5.0%, with an average of 4–12 months depending on the stage of disease, patients’ age and histopathologic subtype [4,5].

From a pathophysiological perspective, malignant pleural mesothelioma originates from pleural mesothelial cells that rapidly spread to the diaphragm, pericardium and lungs [1]. Almost all pleural mesotheliomas are diffuse, and by the time of diagnosis, cancer cells have usually invaded many tissues [6]. For this reason, surgery is often impractical because of the metastatic nature of the carcinoma, as well as the high risks of complications and treatment failure [7].

The only treatment option in patients with unresectable pleural mesothelioma is chemotherapy, with pemetrexed+cisplatin administered first-line and considered the standard of care [8]. Indeed, some studies have demonstrated improved quality of life and favourable clinical outcomes, such as overall survival (OS) and progression-free survival (PFS) [9,10]. In patients without tumour progression, the treatment regimen should be continued for up to six cycles, while in subjects with unacceptable toxicity due to adverse events, pemetrexed+carboplatin could be a possible alternative [11].

In recent years, new treatments have been proposed for this clinical indication, representing a potential therapeutic advancement [12,13,14,15,16,17,18,19]. They include nivolumab plus ipilimumab, bevacizumab plus pemetrexed plus cisplatin, chemotherapy plus pembrolizumab, ONCOS-102 plus pemetrexed plus cisplatin/carboplatin and cediranib plus pemetrexed+cisplatin with maintenance with cediranib. However, there are no current studies aimed at summarizing the efficacy of the available therapeutic options or comparing these new combinations with the standard of care. In 2022, we conducted a preliminary analysis of the four published clinical trials, [20] but in the meantime, more studies have been conducted on the topic.

The aim of this paper was to summarize the current state of the art and provide new evidence on the clinical efficacy of available or proposal first-line treatment options for patients with unresectable pleural mesothelioma. In particular, we reconstructed individual patient data (IPD) from survival curves and then performed indirect analyses among published clinical trials. For this purpose, we used an innovative artificial intelligence tool (the IPDfromKM or Shiny method [21,22]) published in 2021. From 2022 to 2024, this technique were increasingly used especially, but not exclusively, in oncology and cardiology to generate new clinical evidence [23,24,25,26,27]. The reliability and validity of the IPDfromKM method were recently confirmed in the study by Rogula and co-workers, who demonstrated the overlap between the original and reconstructed Kaplan–Meier curves obtained with the artificial intelligence tool [28]. On the other hand, a limitation of this technique is that it generally does not allow for the assessment of covariates that may potentially influence patient outcomes.

## 2. Materials and Methods

### 2.1. Study Design and Literature Search

We performed a systematic literature review by searching two databases (Medline, via PubMed and Scopus). The aim was to identify randomized controlled trials (RCTs), either phase II or phase III, that tested the efficacy of new agents (“novel treatments”) when given as first line in patients with unresectable pleural mesothelioma. The final search was conducted on 5 December 2024. The search term was constructed as follows: “mesothelioma[titl]” with filters on RCTs. Article selection was performed in accordance with the PRISMA algorithm [29].

### 2.2. Inclusion Criteria

Only RCTs conducted on previously untreated patients with unresectable mesothelioma, written in English, were eligible. We included all the RCTs that tested the effects of a combination or a single-agent treatment on OS, using a standard of care in the control group. Since the method of our analysis aimed at reconstructing IPD, another inclusion criterion was the availability of a Kaplan–Meier graph comparing the intervention and control groups based on the OS endpoint.

### 2.3. Data Analysis

We firstly selected OS as the endpoint of the statistical analysis and then performed an indirect comparison between each of the six intervention arms vs. the six control arms (pemetrexed+cisplatin or chemotherapy) pooled together. The hazard ratio (HR), with the 95% confidence interval (CI), was the parameter for testing the superiority of each of the 6 experimental treatments vs. standard of care (pemetrexed+cisplatin). In addition, the six control arms of the included trials were subjected to an assessment of cross-trial heterogeneity, which was based on Wald’s test and the likelihood ratio test. To perform these analyses, we used three statistical packages (“survival”, “survminer”, “survRM2” and “readxl”) of the R-platform (version 4.3.2) [30]. Finally, since the oldest of the six included trials compared pemetrexed+cisplatin vs. cisplatin alone, the arm treated with pemetrexed+cisplatin was pooled with the other 5 control arms given pemetrexed+cisplatin. Then, the arm treated with cisplatin alone was pooled with the experimental arms of the other 5 trials. This allowed us to minimize the heterogeneity of our clinical material by referring to the standard of care (pemetrexed+cisplatin) in our 6 overall survival comparisons.

### 2.4. Reconstruction of Individual Patient Data from Kaplan–Meier Curves and Statistical Analysis

Our analysis, performed with the online version of the IPDfromKM method, included a first phase in which the graph of each Kaplan–Meier curve was digitized using Webplotdigitizer (version 4 online; https://apps.automeris.io/ (accessed 10 August 2024)) and a second phase in which the IPDfromKM algorithm [22] reconstructed individual patient data, separately for each curve, from the x-y coordinates deriving from the digitized KM curves (software version 1.2.3.0; https://www.trialdesign.org/one-page-shell.html#IPDfromKM (accessed 10 August 2024)). Once these databases of reconstructed patients were created, indirect comparisons were made between the experimental treatments and the standard of care, using the same statistical tests (e.g., HR estimated by Cox’s multiple regression model) as in clinical trials based on “real” patients.

## 3. Results

**Figure 1:**
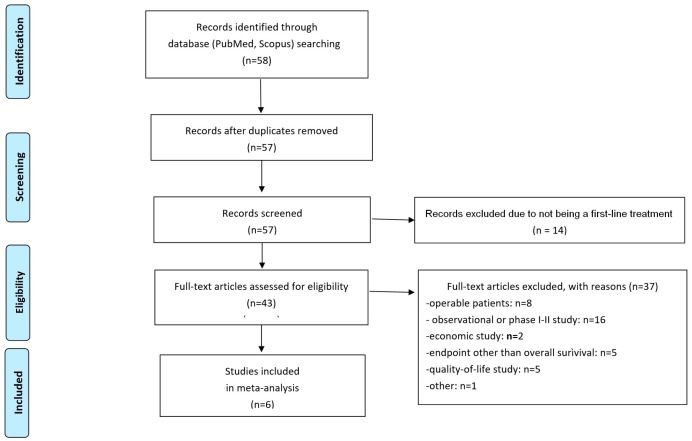
PRISMA flowchart of the literature search. The keyword used for the initial search in the two databases was “mesothelioma [title]” combined with “randomized control trial” as selection term.

Six RCTs [12,13,14,15,16,17] were identified from the literature search. Figure 1 shows the selection process of the included trials according to the PRISMA algorithm. These six trials were identified by searching PubMed. The subsequent search in the Scopus database did not identify any further RCTs for inclusion in our analysis.

The characteristics of these six trials are presented in Table 1 and Table 2. The six treatments tested in these trials included: (1) nivolumab+ipilimumab; (2) bevacizumab+pemetrexed+cisplatin; (3) pembrolizumab+chemotherapy; (4) ONCOS-102 plus pemetrexed plus cisplatin/carboplatin; (5) cediranib plus pemetrexed+platinum; (6) cisplatin alone. In the control arms, the treatment was pemetrexed+cisplatin with the only exception of chemotherapy given to the controls in the trial by Chu et al. (2023) [14]. As previously pointed out, the arm treated with pemetrexed+cisplatin in the study by Vogelzang et al. [17] was grouped with the arms treated with pemetrexed+cisplatin in the other five trials. Regarding the randomized trial by Ponce et al. [15], the treatment arm included ONCOS-102, which is an oncolytic adenovirus expressing granulocyte-macrophage colony-stimulating factor. Since the trial enrolled both pre-treated patients and treatment-naïve patients, only the latter group, consisting of a very small number of patients, met the inclusion criteria of our analysis.

To reconstruct individual patient data, the IPDfromKM technique was applied to the 12 Kaplan–Meier curves reported in these six trials (Table 1). The analysis of reconstructed OS curves was then carried out according to standard survival statistics; indirect comparisons between different treatments were evaluated according to HRs. The six experimental arms of these analyses are shown in Table 2. As control groups for our indirect comparisons, we considered the five arms treated with pemetrexed+platinum and the arm treated with chemotherapy from the trial by Chu et al. (2023) [14]; these six arms were pooled to generate a single Kaplan–Meier curve of 1020 controls.

**Figure 2:**
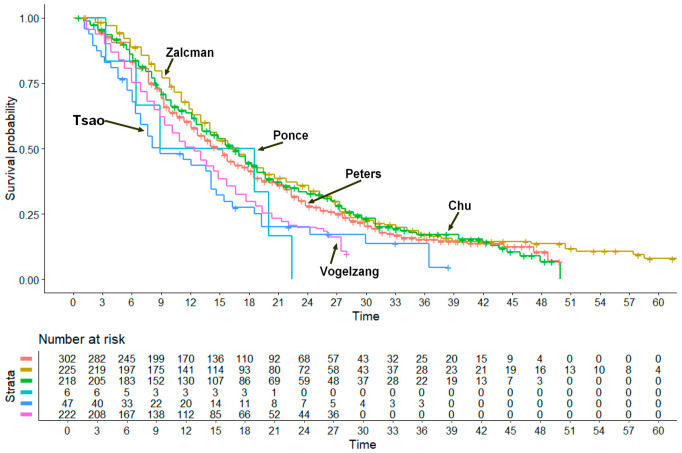
Kaplan–Meier survival curves obtained by reconstructing individual patient data from 6 patient cohorts published in the included trials. The 6 curves refer to the 5 control arms treated with pemetrexed+cisplatinum and one control arm treated with chemotherapy alone in the trial by Chou et al., 2023 [14] (see Table 2). Figure 3 describes the results of our main analysis, in which each of the 6 experimental arms was compared with the 6 control arms pooled together. The HRs for the comparisons of each of the experimental treatment vs. the 6 control arms pooled together are shown in Table 2.

Among the six control arms (Figure 2), the level of cross-trial heterogeneity was significant (likelihood ratio test = 32.6 on 5 df, *p* < 0.001; Wald test = 34.23 on 5 df, *p* < 0.001). However, it should be noted that the first four trials reported in Table 1 and Table 2 (Peters et al., 2022 [12]; Zalcman et al., 2016 [13]; Chu et al., 2023 [14]; Ponce et al., 2023 [15]) showed no significant heterogeneity (data not shown). By contrast, the last two trials in Table 1 and Table 2 (Tsao et al., 2019 [16] and Vogelzang et al., 2003 [17]) were those determining the significant level of overall heterogeneity mentioned above. On the one hand, this can in part be explained by the fact that the trial by Vogelzang et al., 2003 [17], conducted more than 20 years ago, had a worse survival pattern compared with the first four trials, which are more recent; on the other hand, the performance of combining cediranib with other agents, studied in the trial by Tsao et al., (2019) [16], was particularly poor, as confirmed by the 47 controls shown in Figure 2.

With the exceptions of cisplatin alone and cediranib plus pemetrexed+cisplatin, the remaining four experimental arms showed very similar values of median OS, around 18 to 21 months. This indicates that these treatments determine an improvement in OS of slightly more than 3 months.

If these indirect comparisons are examined in more detail, three of the new combination treatments (nivolumab plus ipilimumab, bevacizumab plus pemetrexed plus cisplatin and chemotherapy plus pembrolizumab; see Table 2) determined a significant improvement in overall survival compared with pemetrexed plus cisplatin, whereas cisplatin alone was confirmed to be significantly worse compared with pemetrexed plus cisplatin. Likewise, the two remaining treatments (ONCO-102 plus pemetrexed plus cisplatin or carboplatin and cediranib plus pemetrexed+cisplatin) did not differ from pemetrexed plus cisplatin. Finally, three above-mentioned new combination treatments that fared best in the overall ranking did not differ with one another in terms of overall survival.

## 4. Discussion

The main finding emerging from our analysis is that, in comparison with pemetrexed+ cisplatin, three of the novel treatments proposed as first line (i.e., nivolumab plus ipilimumab, bevacizumab plus pemetrexed plus cisplatin and chemotherapy plus pembrolizumab) provided a significant incremental benefit in OS compared with the standard of care (pemetrexed+cisplatin in five cases out of six), even though the magnitude of this benefit was quite small (around 3 months). The role of the combination of ONCOS-102 with pemetrexed plus cisplatin or carboplatin as first line remains uncertain due to the very small number of enrolled patients. By contrast, we found a negative survival outcome for the regimen based on cediranib, which was numerically inferior to the six control arms pooled together and significantly inferior to the three treatments mentioned above, which showed the best survival outcomes. Furthermore, our results confirmed the inferiority of cisplatin alone compared with pemetrexed plus cisplatin. Among the three best performing treatments given as first-line options, their limited incremental effectiveness aligns with the observations reported for second-line treatments in unresectable mesothelioma [15]. In the context of mesothelioma, even a three-month extension of survival can be meaningful, especially when baseline outcomes are poor. On the other hand, our results raise the issue of whether these gains justify changes in clinical practice, balancing this improved effectiveness against toxicities and cost considerations. In summary, we acknowledge that a survival gain of about three months, while statistically significant, may still be considered modest clinically, and we emphasize the indirect nature of the findings and the potential need for additional prospective trials to confirm.

Our analysis has strengths and limitations. One strength is the excellent performance of the Shiny method in reconstructing individual patient data from published Kaplan–Meier curves, which is in line with findings from the most recent literature [12,16]. In our study, the quality of the reconstruction of individual patient data performed with the IPDfromKM method is confirmed by the agreement between the original HR values (Table 1) and those based on the reconstructed patient data (Table 2). Similarly, when examining all the Shiny analyses published thus far, especially in oncology [12,31], the median values and HRs estimated from the reconstructed curves of each clinical trial prove to be nearly identical to those originally determined from “real” patients. Another strength is that this strategy of evidence analysis enables the execution of indirect comparisons for new therapeutic questions, which are suggested by the recent literature, particularly when direct comparisons based on “real” head-to-head trials are not available.

As regards the limitations of our analysis, the most obvious one has already been mentioned and is the indirect nature of our comparisons. In fact, these indirect retrospective comparisons do not take into account the contribution of intrinsic differences in the patient cohorts from different studies. In contrast, when randomization is applied, it is well known that these characteristics can be balanced between the two groups under comparison.

In summary, regarding novel first-line treatments for unresectable mesothelioma, our article has presented the current state of the art, in which three treatments are characterized by a significant survival advantage. Although the magnitude of their incremental survival benefit is quite small (about 3 months), these new treatments are likely to replace the current standard of pemetrexed plus cisplatin. On the other hand, further research into other combination treatments that may provide a more clinically relevant survival advantage is warranted.

## Figures and Tables

**Figure 3:**
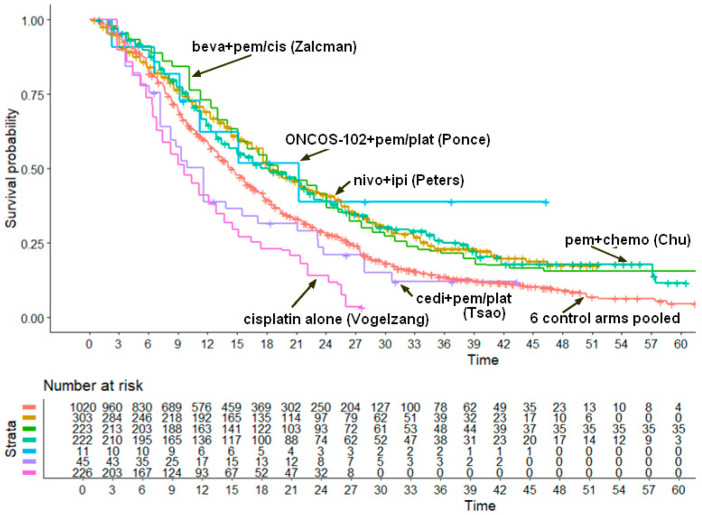
Pooled Kaplan–Meier survival curves obtained by reconstructing individual patient data from 12 patient cohorts published in 6 trials. The curve for the 6 control arms pooled together (in orange) refers to 1020 patients treated with pemetrexed + cisplatin in 5 phase III trials and with chemotherapy alone in the trial by Chu et al., 2023 [14]. The combination of ONCOS-102 with pem+pla did not show a significant improvement in OS (*p* > 0.05). The other 5 curves refer to the 3 treatments (beva plus pem+cis, nivo+ipi and pem+chemo) that performed significantly better than standard of care (control) and to the 2 treatments (cisplatin alone or cedi+pem+plat) that performed significantly worse than the controls (Table 3). Time is expressed in months. Trials are identified based on the first author. Abbreviations: beva, bevacizumab; cis, cisplatin; pem, pembrolizumab; plat, platinum agents; nivo, nivolumab; ipi, ipilimumab; cedi, cediranib.

**Table 1:** Unresectable pleural mesothelioma: characteristics of patients reported in 6 RCT phase III trials.

Study (First Author, Year and Reference)	Gender	Age (Years)	Histology (Number of Patients)	Smoking Status	Performance Status	PD-L1 Level
Peters et al. (2022) [12]	Male: n = 467; female: n = 138;	<65: n = 167; ≥65 to <75: n = 281; ≥75: n = 157;	Epithelioid: n = 455; non-epithelioid: n = 150	Smoker: n = 318; No smoker: n = 249;	ECOG 0: n = 242; ECOG ≥1: n = 363;	PD-L1 < 1%: n = 135; PD-L1 ≥ 1%: n = 451;
Zalcman et al. (2016) [13]	Male: n = 338; female: n = 110;	Median: 65.7; range: 61.3–70.2;	Epithelioid, n = 179; non-epithelioid, n = 44;	Smoker n = 54; No smoker n = 194	ECOG 0–1: n = 433; ECOG 2: n = 15;	NR
Chu et al. (2023) [14]	Male: n = 333; female: n = 107;	Median, 70.9; range: 28.0–88.0;	Epithelioid, n = 349; other, n = 91;	Smoker, n = 245; no smoker, n = 195	ECOG: n = 206; ECOG 1: n = 234;	PD-L1 < 1%: n = 133; PD-L1 = 1%: n = 263;
Ponce et al. (2023) [15]	Male: n = 9; female: n = 22;	Median, 68; range: 36–80;	Epithelioid, n = 24; other, n = 7;	NR	ECOG 0: n = 8; ECOG 1: n = 22;	NR
Tsao et al. (2019) [16]	Male: n = 78; female: n = 14;	Median, 72; range: 46–85;	Epithelioid, n = 69; other, n = 23;	NR	NR	NR
Vogelzang et al. (2003) [17]	Male: n = 365; female: n = 83;	Median, 61; range: 19–85;	Epithelioid, n = 306; other, n = 142;	NR	KPS 70: n = 68; KPS 80: n = 138; KPS 90–100: n = 242;	

Abbreviations: NR, not reported; KPS, Kamofsky performance status.

**Table 2:** Unresectable pleural mesothelioma: information on first-line treatments reported in 6 RCT phase III trials. Endpoint: death for any cause.

Study (First Author, Year and Reference)	Study Design	Intervention vs. Control	Follow-Up (Months)	HR (95% CI) *	Total Number of Events/Patients (n/N)	
					Treatment Group	Control Group
Peters et al. (2022) [12]	RCT	Nivolumab plus ipilimumab vs. pemetrexed plus cisplatin	54	HR = 0.73 (95% CI, 0.61 to 0.87) ^§^	212/303	234/302
Zalcman et al. (2016) [13]	RCT	Bevacizumab plus pemetrexed plus cisplatin vs. pemetrexed plus cisplatin	80	HR = 0.77 (95% CI, 0.62 to 0.95)	164/223	178/225
Chu et al. (2023) [14]	RCT	Chemotherapy ^†^ plus pembrolizumab vs. chemotherapy ^†^	60	HR = 0.79 (95% CI, 0.64 to 0.98)	167/222	175/218
Ponce et al. (2023) [15]	RCT ^§§^	ONCOS-102 ** plus pemetrexed plus cisplatin/carboplatin vs. pemetrexed plus cisplatin/carboplatin	33	NR	6/11	6/6
Tsao et al. (2019) [16]	RCT	Cediranib plus pemetrexed+cisplatin (with maintenance with cediranib) vs. placebo plus pemetrexed+cisplatin (with maintenance with placebo)	40	HR = 0.88 (80% CI, 0.65 to 1.17)	39/45	41/47
Vogelzang et al. (2003) [17]	RCT	Pemetrexed plus cisplatin vs. cisplatin alone	30	HR = 0.77 Median OS, 12.1 vs. 9.3 months	NR/226	NR/222

* These values of HR are those reported by the authors in the original article. ^†^ The chemotherapy in this trial in most cases consisted of pemtrexed plus platinum. ** ONCOS-102 is an oncolytic adenovirus expressing granulocyte-macrophage colony-stimulating factor. ^§^ The paper by Peters et al. [12] did not explicitly report the number of deaths in the two patient groups; this information was therefore estimated from the number of deaths from 0 to 39 months previously reported in the article published by Baas et al. in 2021 [19] (200 and 219 deaths in the two groups, respectively) and by counting the deaths from 40 to 54 months (12 and 15 in the two groups, respectively) according to individual downward steps appearing in the Kaplan–Meier graph published by Peters et al. in 2022 [12]. ^§§^ While the paper by Ponce et al. [15] included 20 patients in the treatment group vs. 6 in the control group, our analysis included only the subgroup of chemo-naïve patients who were 11 in the treatment group and 6 in the control group. Abbreviations: CI, confidence interval; HR, hazard ratio; RCT, randomized controlled trial; OS, overall survival; NR, not reported.

**Table 3:** Values of HR estimated in our main analysis.

First Author, Year and Reference	Treatment Given to the Experimental Arm	Treatment Given to the Control Arm	HR (with 95% CI) Estimated from Reconstrcucted Patients
Peters et al. (2022) [12]	Nivolumab plus ipilimumab	Pemetrexed plus cisplatin	HR = 0.7149 (95% CI, 0.6139 to 0.8326; *p* < 0.001)
Zalcman et al. (2016) [13]	Bevacizumab plus pemetrexed plus cisplatin	Pemetrexed plus cisplatin	HR = 0.7063 (95% CI, 0.6020 to 0.8288; *p* < 0.001)
Chu et al. (2023) [14]	Chemotherapy plus pembrolizumab	Chemotherapy	HR = 0.7297 (95% CI, 0.6170 to 0.8631; *p* < 0.001)
Ponce et al. (2023) [15]	ONCO-102 plus pemetrexed plus cisplatin or carboplatin	Pemetrexed plus cisplatin	HR = 0.5853 (95% CI, 0.2622 to 1.3067; *p* = 0.191)
Tsao et al. (2019) [16]	Cediranib pluspemetrexed+cisplatin (with maintenance with cediranib)	Pemetrexed plus cisplatin	HR = 1.2196 (95% CI, 0.8774 to 1.6953; *p* = 0.237) ^§^
Vogelzang et al. (2003) [17]	Cisplatin alone	Pemetrexed plus cisplatin	HR = 1.7657 (95% CI, 1.5192 to 2.0523; *p* < 0.001) ^§§^

The median OS in the 1020 controls pooled together was 14.6 months (95% CI, 13.6 to 15.6; n = 1020). In the 6 treatment groups, medians of OS were the following: nivolumab plus ipilimumab: 18.29 months (95% CI, 17.63 to 21.9); bevacizumab plus pemetrexed plus cisplatin: 19.13 months (95% CI, 17.15 to 23.1); chemotherapy plus pembrolizumab: 18.26 months (95% CI, 15.01 to 22.1); ONCOS-102 plus pemetrexed plus cisplatin or carboplatin: 21.27 months (95% CI, 11.19 to not computable); cediranib plus cisplatin-pemetrexed: 11.58 months (95% CI, 8.24 to 17.1); cisplatin alone: 9.67 months (95% CI, 8.25 to 11.2). ^§^ The reciprocal of this HR is 0.8199 (95% CI, 0.5899 to 1.1397). ^§§^ The reciprocal of this HR is 0.5663 (95% CI, 0.483 to 0.6582). Abbreviations: CI, confidence interval; HR, hazard ratio.

## Data Availability

The files saved in the format of the IPDfromKM software are available from the corresponding author upon request.
